# Issues and Challenges in the Application of the IEUBK Model in the Health Risk Assessment of Lead: A Case Study from Blantyre Malawi

**DOI:** 10.3390/ijerph18158207

**Published:** 2021-08-03

**Authors:** Wells Utembe, Mary Gulumian

**Affiliations:** 1 Toxicology and Biochemistry Department, National Institute for Occupational Health and National Health Laboratory Service, Johannesburg 2000, South Africa; maryg@nioh.ac.za; 2 School of Public Health, University of the Witwatersrand, Johannesburg 2000, South Africa; 3 Molecular Medicine and Haematology, University of the Witwatersrand, Johannesburg 2000, South Africa; 4 Water Research Group, Unit for Environmental Sciences Management, North West University, Potchefstroom 2351, South Africa

**Keywords:** lead, exposure, biokinetic modelling, children

## Abstract

The risk assessment of lead (Pb) requires the use of biokinetic models to translate measured concentrations of Pb in food and environmental media into blood lead (BPb). The aim of this study was to assess the applicability of the Integrated Exposure Uptake Biokinetic (IEUBK) model in the health risk assessment of Pb among children in Blantyre. Children (152) aged 1–6 years were recruited into this cross-sectional study, and foods, house dust, playground soil, water, and venous blood (1 mL) were collected and analyzed for Pb. A seven-day food frequency questionnaire (FFQ) was used to collect food consumption data. The concentrations of Pb ranged from 0.01 to 3.3 mg/kg in food, 2.3 to 265 mg/kg and 1.5 to 482 mg/kg in house dust and playground soil, respectively, as well as 2.0 µg/dL to 50.4 µg/dL and 6.8 to 39.2 µg/dL for measured and predicted BPb, respectively. Various statistical tests indicated less than satisfactory agreement between measured and predicted BPb values. Despite the lack of reliable food consumption data and other limitations, both the predicted and measured BPb values indicate that children in Blantyre are exposed to high levels of Pb, largely through food and soil as a minor source.

## 1. Introduction

Lead (Pb) causes many adverse effects in adults and children, including anaemia [1], neurobehavioral [2] and nephrotoxic effects [3], and hypertension [4]. In children, Pb has especially been widely associated with reductions in intelligence quotience (IQ) and school performance, as well as an increase in violent behavior [5]. While some effects such as anaemia and nephrotoxic effects occur at high exposure levels, the effects on IQ, ability to pay attention, and academic achievement have been shown to occur even at low blood lead (BPb) concentrations [6]. Compared to adults, children are particularly vulnerable to Pb exposure and poisoning because of their greater hand-to-mouth activity, greater absorptive capacity in the digestive tract, and rapid development of the nervous system [7]. Protection of children from exposure to Pb is very important for lifelong good health since the effects of Pb exposure cannot be reversed [6]. 

In Malawi, the regulation on the sale and use of leaded petrol that was promulgated in 2006 is expected to significantly reduce Pb exposure. However, a large body of evidence shows that other common sources and pathways of Pb including Pb-based house paint [8], food [9], water [10], toys [11], lead-laced china ware [12], cosmetics [13], and medicines [14], could also be significant potential sources of exposure to Pb for children in Malawi. Therefore, there is a need to assess exposure to Pb from different sources and its associated risks among various populations in Malawi. 

Exposure to Pb may be assessed using bottom-up approaches, where chemicals are measured in environmental media such as air, water and food, or using top-down or biomonitoring approaches where chemicals are measured in bodily fluids or other specimens. The former approach furnishes information on external exposures and their sources, without providing information on internal doses, while the latter approach provides the actual concentrations of a chemical in specified bodily fluids, tissues, or specimens, albeit without providing information about sources of exposure [15]. Both approaches (i.e., measurement of Pb in environmental media as well as biomonitoring) are used in the exposure and risk assessment of Pb. 

In particular, the risk assessment of Pb in environmental media is used to provide information on the level of contamination of specific media (food, water, air, soil, etc.), which can in turn be converted to potential doses of Pb. This approach requires the comparison of the amount of Pb intake from the environmental media with safe levels of Pb such as the reference dose (RfD), the acceptable daily intake (ADI), or the provisional tolerable weekly intake (PTWI). However, as the WHO withdrew the PTWI for Pb [16] and also since Pb does not have a reference dose [17], health risk assessment of Pb from various environmental media and food requires translation of the Pb intake from these sources into BPb using biokinetic models. The predicted BPb are subsequently correlated with potential health effects that have been observed at various BPb ranges [18].

Only a few biokinetic models have been developed for converting Pb levels in environmental media to Pb levels in human blood or tissues. These models include the United States Environmental Protection agency (USEPA) Integrated Exposure Uptake Biokinetic (IEUBK) model [19], the Carlisle and Wade model [20], the California Department of Toxic Substances Control LeadSpread model [21], the All-Ages Lead model (AALM) [22], and O’Flaherty physiologically based toxicokinetic (PBTK) model [23]. These biokinetic models are developed based on the mechanistic understanding of the processes occurring in the body, and are therefore quite challenging to develop in terms of human, technical, and financial resources. Hence, for developing countries such as Malawi, it is more prudent to assess the applicability of these existing biokinetic models before any attempts to develop local biokinetic models. 

This study used the IEUBK model because it is validated, it is reported to be accurate [24,25,26], and because it was specifically designed to predict BPb concentrations in children between one and six years of age exposed to Pb in their environment. For these reasons, the IEUBK is the most widely used model for the health risk assessment of Pb in children. The model allows the user to input relevant absorption parameters (e.g., the fraction of Pb absorbed from food) as well as intake and exposure rates [27], and has been used in many countries for different applications. For example, the IEUBK model could be used to predict BPb values resulting from exposure to Pb from different sources [28,29,30], to estimate the contribution of a source of Pb (e.g., tap water) to BPb [28,29], to determine exposure routes [31], as well as to estimate the concentration of Pb in soil in remediation that would result in a predetermined percentage of children (such as 10%) not having BPb above 5 µg/dL [31,32,33,34]. 

Despite its numerous applications in many countries, the IEUBK model has not yet been used in Malawi or in any other country in Africa, prior to this study. Therefore, it was necessary to first evaluate the applicability of the model in Malawi by assessing the agreement, if any, between the measured and predicted BPb values. Indeed, the applicability of the IEUBK has been assessed in a number of countries including, among others, the USA [32], Belgium [31], China [35,36], and Australia [37]. The study approach in these countries has largely been based on the comparison of the predicted BPb data with the BPb data measured in cross-sectional studies. Similarly, in this study, BPb data was obtained from children in Blantyre through a descriptive cross-sectional epidemiological study and was compared with BPb predicted from the model based on Pb levels in various environmental media. Furthermore, a risk assessment was conducted based on these data. However, since risks are assessed by correlating the predicted BPb values with potential health effects that are observed at various BPb ranges [38], the risk assessment procedure for Pb does not follow the typical risk assessment process for carcinogenic and non-carcinogenic substances, which utilize cancer slope factors and reference doses, respectively. 

## 2. Methodology

### 2.1. Study Setting and Study Population

Blantyre City is divided into six health catchment areas, which are served by six main health centers. These areas are Machinjiri, Chilomoni, Ndirande, Limbe, Zingwangwa, and Bangwe. Blantyre has many residential, commercial, and industrial areas, with some of the residential areas located in close proximity to industrial sites. However, there are no mining activities or Pb smelters close to Blantyre City, and there are no known industrial activities that can result in occupational and/or environmental exposure to Pb.

The study population included children between 1–6 years of age, who had lived at the residence for at least six months prior to enrolment. The population of the age group 1–6 years in Blantyre can be estimated to be over 100,000 [39]. Children that were still under breast-feeding were excluded from the study. 

### 2.2. Recruitment of Children

Ethics approval was obtained from the University of the Witwatersrand Committee for Research on Human Subjects (No. M120662) as well as from Malawi College of Medicine Research Ethics Committee (COMREC) (No. P.09/12/1282). The sample size was calculated based on Pb exposure from Botswana [40] due to its similar socio-economic characteristics and climate. Using statistical parameters from this study and an equation for calculating sample sizes for prevalence studies [41], the minimum sample size was calculated to be 120.

(1) $$n=\frac{Z_{\alpha }\sigma^{2}}{d^{2}}.\;$$

where *n* = number of children.

*Z*_α_ = standard normal deviate corresponding to a 2-sided level of significance of 5% = 1.96.

σ = standard deviation of Pb level from previous study = 5.6 µg/dL.

*d* = level of precision = 1 µg/dL.

*n* = 120.

In order to account for an estimated refusal or non-response rate of 50%, the targeted number of children in the study was 240, comprising of 40 children from each of the 6 health catchment areas of Blantyre. Therefore, after introducing the project to community leaders, 40 names of eligible children were randomly chosen from the community nurses’ and health surveillance assistants’ (HSAs) register in each health catchment area. Subsequently, study information sheets written in both English and the vernacular Chichewa were given to the children’s parents or guardians. Only individuals that consented to take part in the study were enrolled. 

### 2.3. Sampling and Sample Collection 3.1. Blood

One millimeter venous samples of whole blood were drawn into Vacutainer tubes with techniques designed to ensure minimal Pb contamination, as is recommended in the literature [42]. These samples were stored at 4–6 °C at the College of Medicine laboratory awaiting transportation to Lancet Laboratories in South Africa for analysis.

#### 2.3.2. Food and Water

Samples of the most commonly consumed foods as determined by the food frequency questionnaire (FFQ) were acquired from the market in the health catchment area concerned, as recommended in the WHO guidelines [43]. Wherever necessary, the food was prepared by a few participating women from each catchment area using the most common methods of preparation. The foods were then stored at −20 °C at the College of Medicine cold room. 

A water (250 mL) sample was drawn from the homes of the participants at any random time of the day, as is commonly practiced in the literature [44]. The samples of water were then stored at −20 °C at the College of Medicine cold room.

#### 2.3.3. House Dust and Soil

Floor dust samples were collected from the children’s bedroom wherever possible or in the lounge, using a broom or brush from each particular home. Use of broom or brush for sampling dust is a method that is found in the literature [45] together with wipe sampling methods and use of vacuum cleaners [46,47]. In some cases, samples of dust were also obtained from the school that the children were attending. The dust samples were not touched with bare hands to avoid contamination, as is recommended by the US Department of Housing and Urban Development (HUD) [48].

Two samples of surface soil from the children playground at home were collected into a sample container (60 mL bottles) by a scoop. In some cases, samples of soil were also collected from the playground at the school that the children were attending. 

#### 2.3.4. Food Consumption Data

Food consumption data was collected using a seven-day food frequency questionnaire (FFQ) that was adopted from the Birth-to-Twenty cohort study and the Malawi Second Integrated Household Survey (IHS) questionnaire [49]. Food conversion factors were adopted from the South African Medical Research Council (MRC) Food Photo manual [50] and the Malawi Third IHS 2010/11 Data [51].

### 2.4. Laboratory Analysis of Lead in Various Samples 

Blood samples were analyzed at Lancet Laboratories in Johannesburg, South Africa, whereas dust, soil, food, water, paint, and toy samples were analyzed at Protechnik Laboratories (a Division of Armscor SOC Ltd.in Pretoria, South Africa, both being accredited commercial laboratories that participate in national and international quality control programs. Quality assurance was maintained by use of these certified laboratories as well as appropriate certified reference materials. 

#### 2.4.1. Lead in Blood

Whole blood samples were diluted ten times by adding 100 µL of each blood sample to 900 µL of diluent (10% Triton X-100). Analysis was performed on a Varian SpectrAA 220Z Graphite Furnace Atomic Absorption Spectroscopy (GFAAS). The instrument was calibrated with calibration standards prepared in sheep blood for matrix matching. The detection limit (three times standard deviation of all blank samples) for Pb in whole blood was 1 μg/dL, while the uncertainty of reading was 14.5%.

#### 2.4.2. Lead in Food and Water

A known weight (approximately 2 g) of the food sample was dry-ashed at 420 °C. Ashed samples were dissolved in 10 mL 0.25% HNO_3_. The Pb concentrations were then determined by ICP-MS. Water samples were acidified before determination of Pb using an ICP-MS method based on NIOSH 7300 and ISO 15202-3.

#### 2.4.3. Lead in House Dust and Soil

House dust and soil samples were digested in nitric and perchloric acids, and after filtration, the solutions were analyzed for Pb using an ICP-MS method based on National Institute of Occupational Safety and Health (NIOSH) 7300 and ISO 15202-3.

### 2.5. Data Processing and Analysis

#### 2.5.1. Data Entry 

Data were entered into Microsoft Excel 2007 spreadsheets, cleaned, and then transferred to STATA version 12 statistical package spreadsheets for analyses. Descriptive statistics including means and standard deviations were calculated for all the Pb concentrations. 

#### 2.5.2. Prediction of Blood Lead from Food, Water, House Dust, and Soil Using the IEUBK Model

In order to match the laboratory analysis of Pb in food, which were based on dry weight, moisture content was corrected using the following Equation (2) [52]:

(2) $$IR_{Dry}=IR\;\frac{\left(100-M\right)}{100}$$

where *IR_Dry_* is the average consumption rate of the food item on a dry basis, *IR* is the average consumption of the food item as given in the FFQ and *M* is the moisture content of the food. 

The calculated *IR_Dry_* could then be used to calculate the dietary intake (dose), which was subsequently used in the IEUBK model. For food, the IEUBK model default bioavailability of 50% (as recommended for the model) and a 31% value from the literature [53] were used in the present study, while for soil a default bioavailability of 30% was used. 

#### 2.5.3. Assessment of Model Performance

Model performance was assessed by calculating the modelling efficiency (ME), the Nash-Sutcliffe efficiency (NSE), the Root Mean Deviation (RMD), the ‘95% limits of agreement method’, and by using the paired student’s t test. Since each one of model performance criteria emphasizes on different aspects of model performance, it is important to use a combination and not just one criterion. 

ME compares the efficiency of the model to the efficiency of describing the data using the mean of the observations. In other words, the ME indicates whether the model describes the data better than simply the average of the measurements. The ME is calculated as shown in Equation (3):

(3) $$ME=\frac{\sum_{i=1}^{n}{\left(O_{i}-O_{Av}\right)}^{2}-\sum_{i=1}^{n}{\left(P_{i}-O_{i}\right)}^{2}}{\sum_{i=1}^{n}{\left(O_{i}-O_{Av}\right)}^{2}}$$

where *P_i_* is the predicted BPb value, *O_i_* is the observed or measured BPb value, *O_Av_* is the average of the observed values, and *n* is the number of values [54]. The values of *ME* range from −1 to +1, where a positive value of *ME* indicates that the predicted values provide a better trend than the mean of observed values, while a negative value of *ME* indicates that the predicted values do not describe the trend better than the mean of observed values [55].

The NSE is a “statistic that determines the relative magnitude of the residual variance compared to the measured data variance” [43]. It can be computed using Equation (4):

(4) $$NSE=1-\frac{\sum_{i=1}^{n}{\left(O_{i}-P_{i}\right)}^{2}}{\sum_{i=1}^{n}{\left(O_{i}-O_{av}\right)}^{2}}$$

where *O_i_* is the observed or measured BPb value at a particular place or time *i*, *P_i_* is the predicted BPb value at a particular place or time I, and *O_Av_* is the average of the observed values. *NSE* indicates how well the plot of measured versus predicted data fits the ‘1:1 line’. *NSE* values range between −∞ and 1.0, with an optimal value of 1, where “values between 0 and 1 are generally viewed as acceptable levels of performance, whereas values < 0.0 indicates that the mean observed value is a better predictor than the simulated value, which indicates unacceptable performance” [56].

The RMD is a parameter that evaluates bias in the model, with values close to 0, indicating absence of bias [35]. It can be calculated using Equation (5):

(5) $$RMD=\frac{100}{O_{i}}\overset{n}{\underset{i=1}{\sum }}\frac{P_{i}-O_{i}}{n}$$

The 95% agreement method is a simple statistical method in which the difference between measurements on the same individual by two methods is plotted against the mean of the two measurements. In this method, the 95% of differences between measurements by two methods are expected to lie between the mean difference ± 2 standard deviations [57]. Finally, the paired student’s *t* test determines if there is a significant difference in the means of independent paired data.

#### 2.5.4. Assessment of Potential Effects of Lead

Potential health effects of Pb were assessed in terms of measured BPb concentrations using cut-off points or thresholds for BPb concentrations for various health outcomes, which include reduction of IQ (5 µg/dL), gastro-intestinal effects (60 µg/d)L, and anaemia (70 µg/dL) [58]. 

## 3. Results

### 3.1. Concentrations of Lead in Food, Water, House Dust, and Soil

The concentrations of Pb in food ranged from 0.01 in chicken to 3.3 mg/kg in potato chips. All water samples, on the other hand, contained Pb in amounts that were below the detection limit of 0.000018 mg/L. The concentrations of Pb in house dust ranged from 2.3 to 265 mg/kg, with an outlying figure of 17,179 mg/kg. Apart from the latter outlying figure, the concentrations of Pb in house dust in most homes were much lower than the USA limit of 400 mg/kg for Pb in soil in playgrounds. The concentration of Pb in soil ranged from 1.5 to 482 mg/kg, with only one sample of soil containing Pb above the limit of 400 mg/kg. Therefore, it can be concluded that in general, the concentrations of Pb in house dust and playground soil in many homes in Blantyre were very low. 

### 3.2. Predictions by the IEUBK Model in Comparison with Measured Blood Lead

**Figure 1:**
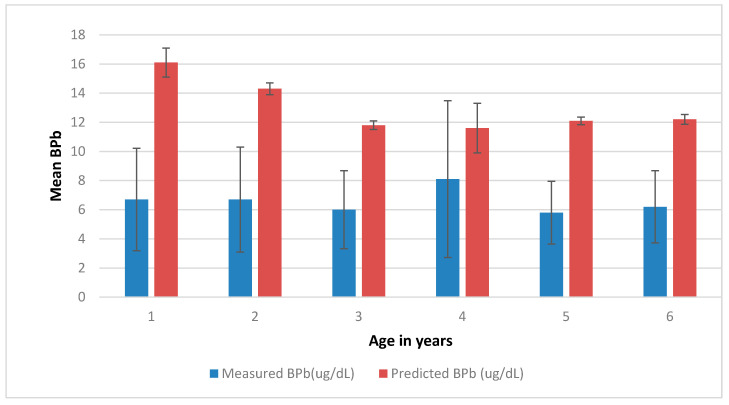
Comparison of the geometric means predicted and mean observed BPb against age (for 50% food bioavailability and a default 30% soil bioavailability).

Since Pb in air was not measured in this study and also since the concentrations for Pb in water were below detection limit, it was only possible to use the Pb levels assessed in food, soil, and house dust. Using these latter values, in addition to the default and published values for bioavailability of Pb from these sources, it was possible to predict the BPb levels resulting from exposure to Pb from the aforementioned three sources. For example, using the default bioavailability value for Pb in food (50%) and a default bioavailability value for Pb in soil (30%), the obtained predicted values of BPb ranged from 10.5 to 39.2 µg/dL, with a geometric mean of 12.5 µg/dL, compared to the measured BPb values, which ranged from 2.0 to 50.4 µg/dL, with a geometric mean of 6.5 µg/dL. The comparison of the geometric means of predicted BPb with the geometric means of observed BPb segregated by age of children is presented in Figure 1. 

It is important to note that the IEUBK model is designed to predict BPb values below 30 µg/dL for individuals, as well as geometric means for populations. Indeed, since BPb values above 30 µg/dL would be considered very high even among occupationally exposed adults, it was not envisaged before the initiation of the study that children would have BPb values above this value. Therefore, two BPb values above 30 µg/dL (37 and 52 µg/dL) were removed in the calculation of the averages, which also removed the skewness of the data brought by values above 30 µg/dL. However, it needs to be pointed out that since only two children had BPb levels above this limit 30 µg/dL, the removal of these figures is expected to have minimal or negligible impacts on the results. 

The ME of −2.24, NSE of −3.30, RMD of 88, and the students’ *t*-test have indicated poor agreement between the predicted and measured BPb values, and that the measured mean was a better description than the values predicted by the model. Subsequently, it could be said that there was significant difference between measured and predicted BPb values when a default bioavailability of 50% for Pb in food and 30% for Pb in soil were used, where the predicted values were on average two-fold higher than the measured values. 

**Figure 2:**
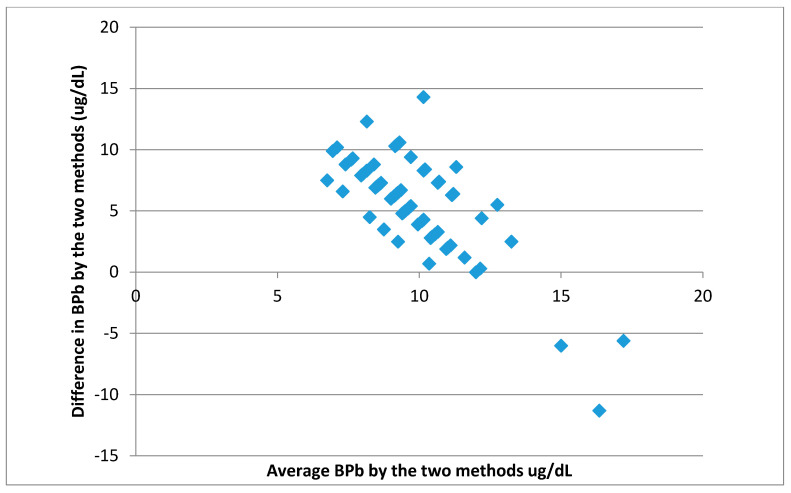
A plot of the average of the measured and predicted BPb against the differences between predicted and measured BPb values (for 50% food bioavailability).

Furthermore, using the 95% agreement method, the averages of the measured and predicted BPb values were plotted against the differences between predicted and measured values for each individual (Figure 2 below). The differences between predicted and measured values have a mean of 5.96 µg/dL and a standard deviation of 3.65. Therefore, as the 95% limits are 5.96 ± 1.96 × 3.65 (i.e., 13.1 and −1.11), it can be concluded that for 95% of children, prediction by the IEUBK model would be between about 1 µg/dL less and about 13 µg/dL higher than measured values [59]. Since the critical value for BPb in children is only 5 µg/dL, the model would tend to over-predict most BPb values when 50% dietary Pb bioavailability is used. This over-prediction is consistent with the results displayed in Figure 1 above. 

**Figure 3:**
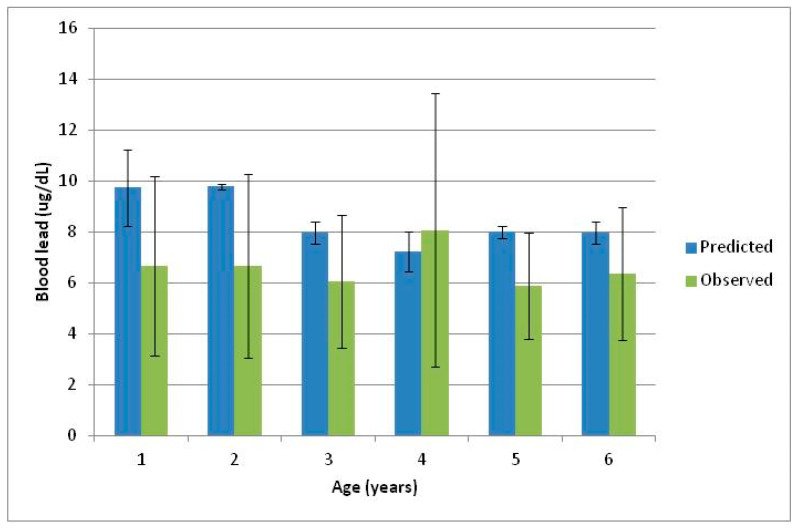
Comparison of the geometric means predicted and mean observed blood Pb against age (for 31% food Pb bioavailability and a default 30% soil Pb bioavailability).

On the other hand, using a bioavailability of 31% for Pb in food obtained from the literature and a default bioavailability of 30% for Pb in soil, the predicted BPb values obtained have ranged from 6.8 to 33.9 µg/dL, with geometric mean of 8.30 µg/dL. In comparison, measured BPb values ranged from 2.0 to 50.4 µg/dL, with a geometric mean of 6.5 µg/dL. The comparison of the geometric means of predicted BPb with the geometric means of observed BPb segregated by age of children is provided in Figure 3.

In this instance as seen in Figure 3, the NSE of −0.3, and a paired *t*-test values have indicated poor agreement between the predicted and measured BPb values. On the other hand, an RMD value of 25.7 indicated slight bias in the model and an ME value of +0.49 has shown that the predicted values are better indicators of BPb levels than the mean of measured BPb. As such, the ME and RMD values have provided an acceptable agreement between the two sets of values, where the predicted values were on average only 1.3-fold higher than the measured BPb values. 

**Figure 4:**
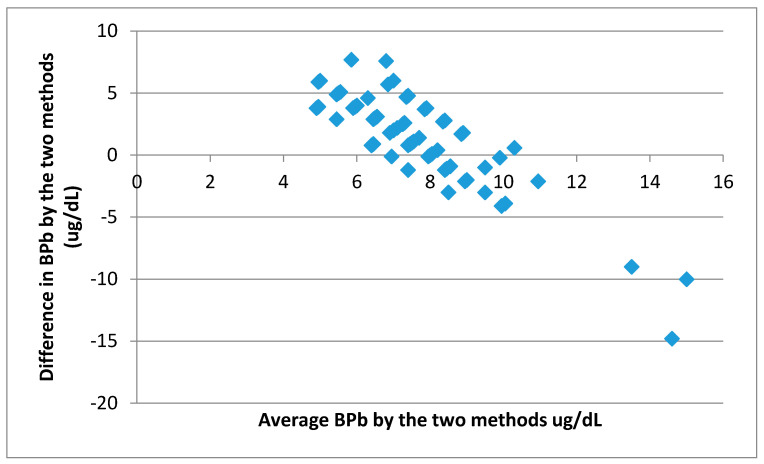
A plot of the average of the measured and predicted BPb against the differences between predicted and measured BPb values (for 31% food Pb bioavailability and a default 30% soil Pb bioavailability).

The differences between predicted and measured values have a mean of 1.74 µg/dL and a standard deviation of 3.44 (Figure 4). Therefore, as the 95% limits are 1.74 ± 1.96 × 3.44 (i.e., −5.0 and 8.48), it can be concluded that for 95% of children, prediction by the IEUBK model would be between about −5 µg/dL less and about 8 µg/dL higher than measured values. Predictions of 5 µg/dL less or 8 µg/dL higher than measured values may result in misallocation of a child’s BPb, especially since the BPb level of action is only 5 µg/dL. However, there is much better agreement between predicted and measured BPb than in the case when 50% dietary Pb bioavailability was used, which indicates that the value of bioavailability used in the model can have a significant impact on the level of agreement between predicted and measured BPb values. 

### 3.3. Potential Health Effects of Blood Lead 

There were 152 subjects (82 males and 70 females), aged between one to six years (average 4.07 ± 1.59). Measured BPb values ranged from 2.0–50.4 µg/dL, with an average of 6.9 ± 5.3 µg/dL. Approximately, 72% of the children had high BPb, i.e., BPb ≥ 5 µg/dL and 22.8% had BPb ≥ 10 µg/dL. These results show that almost 72% of the children may be at risk of suffering from IQ reduction. On the other hand, none of the children had BPb levels above 60 μg/dL, and hence the risk of suffering from Pb-induced anaemia and gastro-intestinal effects were non-existent.

Depending on the bioavailabilities used, BPb ranged from approximately 7 to 15.5 µg/dL, indicating that the model would predict that all the children would be at risk of suffering from IQ reduction. 

## 4. Discussion 

Statistical analyses performed in this study have shown different levels of agreement between measured and predicted BPb depending on the bioavailability used. The IEUBK model was validated using some default bioavailability values and their use is recommended. Therefore, the present study used these default bioavailability parameters. However, it is important to note that these values were obtained from studies in America and Europe, and therefore may not be appropriate for all children and for every site-specific application such as in Malawi. Bioavailability is known to be affected by site-specific soil properties such as soil type, pH, and cation-exchange capacity [60], as well as nutritional status of the subjects [61] and the type of food matrix [62]. However, using default bioavailability values, close agreement was achieved in China [35], while the model was over-predictive in Mexico [63], USA [32], and Belgium [31]. Better agreement between measured and predicted BPb values are anticipated if site-specific bioavailabilities were utilized, as was achieved in Kazakhstan [24]. Since the determination of bioavailabilities require independent and dedicated studies, their determination is highly recommended in any future use of the model in Malawi. 

The less than perfect agreement between measured and predicted data can also be attributed to a number of factors. Firstly, due to lack of more reliable food consumption data, the food consumption data used was obtained using a seven-day FFQ. Although the food consumption data was based on local data, and therefore can be said to more relevant than national data, the data was obtained from only 152 participants, which is much smaller than the sample sizes used in typical food consumption surveys [64]. There was need for food consumption data from independent and dedicated food consumption surveys repeated over time to address issues of intra-and inter-individual variabilities [65]. In addition, since the FFQ method requests participants to recall the foods consumed in the past seven days including types, amounts, and frequency, food intake assessments obtained through this retrospective approach are prone to errors that arise from the use of memory and also from recall bias [66]. In that regard, more reliable food consumption data can be obtained using prospective dietary assessment methods such as duplicate portion studies or food diaries involving larger numbers of participants and covering longer periods of time [67,68]. The prospective food intake assessment methods could not be used in the present study because of cost and logistical reasons. 

In addition to the use of recommended (default) bioavailability values, the study used bioavailabilities from the literature. Despite the lack of agreement between measured BPb and predicted BPb, calculations from the model and the relatively high levels of Pb in food in relation to the high food consumption rates seem to indicate food is the major contributor to the high BPb in children. Indeed, investigations in the literature have also shown that food has been the major contributor to BPb in China (83.4%) [35] and Belgium (over 75%) [31]. Therefore, the present results and those presented in the literature reiterate the importance of food as a major source of BPb, and hence further investigations are recommended to elucidate the origins of high Pb content in food. Dietary Pb originates from environmental sources such as soil, water, and air, as well as food processing, food handling, and food packaging [69]. Environmental Pb food contamination may result from Pb that is strongly adsorbed to soil and organic particles [70]. The Pb can persist for years and be later absorbed in crops through the roots and translocated to aerial parts of the plant. Lead contamination of food can also result from use of contaminated utensils/apparatus for food preparation or storage as well as packaging materials [71]. Therefore, there is a need to investigate the contribution of these sources to dietary Pb in Blantyre Malawi.

The IEUBK model could also show that soil and house dust are minor contributors (11.2%). These results were in agreement with those reported for China (15%) [35], but in disagreement with those reported for Australia (54%) [38]. Indeed, outdoor soil has been reported to be a reservoir of legacy Pb from multiple anthropogenic sources, resulting in contemporary Pb exposure through soil-air dust- home (or school) pathways [72]. Exposure to this legacy soil Pb primarily through re-suspended dust is subject to climatic and seasonal factors such as rainfall, wind, and humidity. 

In contrast to soil, water samples in the present study were shown to contain undetectable amounts of Pb, and hence it could be said that contribution of water to BPb was minimal. 

While the predicted BPb data indicates that all children in Blantyre have high BPB levels (6.8 to 39.2 µg/dL), the measured BPb values have shown that 71.7% of children have BPb ≥ 5 µg/dL and 23% have BPb ≥ 10 µg/dL. Similar investigations conducted using measured BPb values in other countries in the region have also shown high levels of exposure to Pb among children. These have included Botswana, where 31% of the children had BPb levels ≥ 10 µg/dL [40] and South Africa, where 78% of children had BPb ≥ 10 µg/dL [73]. However, it needs to be pointed out that the studies conducted in Botswana and South Africa were conducted soon after the introduction of regulations on leaded petrol in these countries, whereas the present study has been conducted about ten years after the introduction of these regulations. On the other hand, levels of BPb in the present study were much higher than those reported in countries from other regions such as China, where only 1.32% of children had BPb above 5 µg/dL [74]. 

The IEUBK model is designed to predict BPb levels from exposure to Pb from a limited number of external sources including food, water, air, soil, and house dust due to the fact that the intake rates from these external sources are possible to assess. For the same token, it is therefore not possible to use this model to predict BPb from external sources such as cosmetics, children’s toys, (traditional) medicines, and paint as the intake or exposure rates to Pb from these sources are not practically possible to assess. Although exclusion of Pb exposure from these sources has not affected the performance of the model in China [35], significant contributions from these sources may result in poor agreement between measured BPb and BPb predicted from the model, as was observed by von Lindern et al. [32]. In Mexico, the main source of variability was reported to be glazed ceramic pottery [63]. The lead-glazed pottery, direct exposure to Pb from paint chips, toys, cosmetics, and medicine are miscellaneous sources that need to be well-characterized in separate risk assessments of these individual products. 

Although cross-sectional study designs were successfully used in Mexico [63], Belgium [31], Australia [37], and China [35] to assess the applicability of the model in their countries, the present study may have been affected by the use of this study design, since it only captures a snapshot of exposure levels at a particular time, on the assumption that levels of exposure to each individual remain constant with time. Contrary to the assumption of constant environmental Pb concentrations, however, levels of Pb in dust, soil, and food, may vary with time, and thereby affect the cumulative levels of BPb. Indeed, Pb in the blood of each child was affected by the cumulative exposure levels, which were changing with time. These variations may have significantly contributed to the poor agreement between predicted and measured BPb values. In contrast to cross-sectional studies, a longitudinal study design, which captures the variations in exposure levels, may be recommended for future studies, where measurements of Pb in the same participants and their environments may be taken periodically over time. Indeed, in their discussion on the validation strategies for the IEUBK model, the USEPA [75] acknowledged the importance and ‘usefulness’ of longitudinal studies, but also recognized the role of cross-sectional studies because of the time and financial constraints associated with longitudinal studies. It is important to note, however, that while Gulson et al. [76] used longitudinal data in Australia to show good agreement between predicted and measured BPb, use of longitudinal data in the USA by von Lindern et al. [32] resulted in over-prediction of the model. This emphasizes the importance of other factors to the accuracy of the model, in addition to the study design. 

The study did also not consider the contribution of maternal Pb due to lack of data on maternal Pb in Malawi. However, since Pb can easily be transferred through the placenta [77] and through breast milk [78] maternal Pb can be a significant source of Pbin children. In the present study, the effect of maternal Pb was ameliorated by the exclusion of children that were still on breast milk at the time of the study. This notwithstanding, the contribution of the reservoirs of bone Pb that was acquired in utero as well as from breast milk may still have an effect on BPb levels for many years [79,80]. Finally, concentrations of Pb in air were not measured, on the assumption that banning of leaded petrol has reduced its concentrations in air to negligible levels [81]. 

## 5. Conclusions

This study aimed to utilize the IEUBK model to convert the concentrations of Pb in food, water, and environmental media BPb concentrations in children aged between one and six years. There was less than perfect agreement between measured and predicted BPb values in this study because of a number of issues and challenges. Firstly, statistical analyses indicated different levels of agreement between measured and predicted BPb depending on the bioavailability used. The present study used recommended (default) bioavailability parameters from studies in America and Europe, which may not be appropriate for Malawi. The application of the IEUBK in Malawi also needs more reliable food consumption data, which were not available at the time of the study. 

The IEUBK model is designed to predict BPb levels from exposure to Pb from a limited number of external sources including food, water, air, soil, and house dust and not from other sources such as cosmetics, children’s toys, (traditional) medicines, and paint where the exposure or intake rates are impractical or difficult to assess. Significant contributions from these sources would result in poor agreement between measured BPb and BPb predicted from the model. 

The cross-sectional design used in this study may not adequately capture the varying levels of Pb in dust, soil, and food, which affect the cumulative levels of BPb. Therefore, more accurate predictions from the IEUBK model may be expected from longitudinal data, rather than cross-sectional data. 

In the end, the IEUBK model did not yield satisfactory results and its use in Malawi is contingent on proof of its applicability using, among others, site-specific bioavailability values and food consumption data that accounts for inter- and intra-individual variabilities. Nevertheless, both predicted and measured BPb indicate that children in Blantyre are exposed to high levels of Pb. Therefore, further studies are needed to establish the origin of Pb as well as methods for reducing it in all areas of Blantyre. 

## Data Availability

More data on the study can be accessed at http://wiredspace.wits.ac.za/xmlui/bitstream/handle/10539/22245/Thesis%20Wells%20Utembe%20Final%20resubmission%2015%20August%202016.pdf?sequence=1&isAllowed=y (accessed on 8 July 2021).
